# *OsSIZ*2 exerts regulatory influences on the developmental responses and phosphate homeostasis in rice

**DOI:** 10.1038/s41598-017-10274-5

**Published:** 2017-09-25

**Authors:** Wenxia Pei, Ajay Jain, Yafei Sun, Zhantian Zhang, Hao Ai, Xiuli Liu, Huadun Wang, Bing Feng, Rui Sun, Hongmin Zhou, Guohua Xu, Shubin Sun

**Affiliations:** 1 State Key Laboratory of Crop Genetics and Germplasm Enhancement, Key Laboratory of Plant Nutrition and Fertilization in Low-Middle Reaches of the Yangtze River, Ministry of Agriculture, Nanjing Agricultural University, 210095 Nanjing, China; 2 Amity Centre of Nano Biotechnology and Plant Nutrition, Kant Kalwar, NH-11C, Jaipur, 303002 India; 3 Present Address: Provincial Key Laboratory of Agrobiology, Jiangsu Academy of Agricultural Sciences, Nanjing, China

## Abstract

*OsSIZ1*, a small ubiquitin-related modifier (SUMO) E3 ligase, exerts regulatory influences on the developmental responses and phosphate (Pi) homeostasis in rice (*Oryza sativa*). Whether paralogs *OsSIZ1* and *OsSIZ2* are functionally redundant or the latter regulates these traits independent of the former is not known. To determine this, in this study, *OsSIZ*2 was functionally characterized by employing reverse genetic approaches. Although the relative expression of *OsSIZ*2 was spatiotemporally regulated, it showed constitutive expression in root and leaf blade irrespective of Pi regime. Analysis of T-DNA insertion knockout (*ossiz2*) and RNAi-mediated knockdown (Ri1-3) mutants revealed positive influences on growth and developmental responses including yield-related traits. On the contrary, these mutants exhibited negative effects on the concentrations of Pi and total P in different tissues. The relative expression levels of some of the genes that are involved in Pi sensing and signaling cascades were differentially modulated in the mutants. Further, attenuation in the expression levels of *OsSIZ2* in the roots of *ossiz1* and relatively similar trend of the effects of the mutation in *OsSIZ1* and *OsSIZ2* on growth and development and total P concentration in different tissues suggested a prevalence of partial functional redundancy between these paralogs.

## Introduction

The current world population of 7.3 billion is expected to escalate to 9.7 billion by 2050 (www.un.org/en/development). For sustainably feeding such a large population, food production needs to be increased by ~70% (www.fao.org). Rice (*Oryza sativa*) is a staple for almost half of the world′s seven billion people of which ~90% is consumed in Asia alone (www.irri.org/rice-today). The world′s dietary energy supply is contributed maximum by rice (20%), followed by wheat (19%) and maize (5%) (www.research.cornell.edu). Rice is thus an ideal crop for global food security and its increased production and productivity is needed now more than ever.

The essential macronutrient phosphorus (P), a structural component of cellular macromolecules such as phospholipids and nucleic acids, plays a key role in energy-dependent metabolic pathways in plants^1,2^. Inorganic phosphate (Pi) is the preferred form of P taken up by plants of which 80% is immobile and not readily available in rhizospheres^3^. Pi deficiency is common in many of the rice ecosystems, particularly in acid upland soils with high soil P-fixation capacity and adversely affects root development, tillering and flowering^4,5^. Although soils are conventionally enriched with P fertilizer, its excessive usage is uneconomical for sustainable agriculture and also poses a threat to the environment. Therefore, it is imperative to develop plants with higher Pi use efficiency by biotechnological interventions^6,7^. In this context, it would be worthwhile to identify some of the molecular entities that influence the maintenance of Pi homeostasis. These molecular entities can potentially be engineered for generating ‘smart’ plants amenable for soils poor in Pi.

Plants have evolved an array of adaptive responses to circumvent the adverse effects of Pi deficiency^8^. Genome-wide transcriptome analysis of rice (*Oryza sativa*) seedlings grown under different Pi regime has facilitated in identifying spatiotemporal variations in transcript abundance of an array of a functionally diverse group of genes^9–12^. Current advances in RNA-Seq transcriptome analysis has expedited in identifying differently transcribed genes in Pi-deprived rice at single-base resolution^13,14^. Many of these genes are regulated by a repertoire of transcriptional and post-translational events^15^. Post-translational modifications such as sumoylation and ubiquitylation are critical in determining the activity of proteins, which then mediate complex regulatory processes that are important for the functioning of a cell^16^.

Sumoylation involves the attachment of small ubiquitin-like modifier (SUMO) peptide to the target proteins by a specific enzymatic cascade, which alters their activities, localizations and/or abilities to interact with other proteins^16^. In Arabidopsis, SUMO E3 ligase *AtSIZ1* (At5g60410) plays diverse roles in the developmental processes and various biotic and abiotic stresses^17^. *In vitro*, sumoylation of MYB transcription factor *AtPHR1* (At4g28610) by *AtSIZ1* and exaggerated Pi deficiency responses of the mutant *siz1* provided evidence towards its role in regulating both negatively and positively different Pi deficiency responses in Arabidopsis^18^. *AtSIZ1* has also been implicated in negatively regulating Pi deficiency-induced modulation of root architecture by exerting influence on auxin patterning^19^. *OsSIZ1* and *OsSIZ2* are the homologs of *AtSIZ1* in rice, which partially complemented the morphological phenotype of *siz1* in Arabidopsis^20^. This suggested a likely role of *OsSIZ1* and/or *OsSIZ2* in the maintenance of Pi homeostasis. This assumption gained some credibility from a study which demonstrated elevated uptake of Pi in creeping bentgrass by heterologous expression of *OsSIZ1* ^21^. Furthermore, the mutation in *OsSIZ1* triggered elevated concentration of Pi and altered expression levels of several genes involved in the maintenance of Pi homeostasis in *ossiz1* ^22^ and adversely affected the developmental responses of several reproductive traits^23,24^. This raised a pertinent question whether paralogs *OsSIZ1* and *OsSIZ2* are functionally redundant or the latter exerts independent regulatory influences on the various developmental responses and/or the homeostasis of Pi.

Here, T-DNA insertion knockout mutation and RNAi-mediated suppression were employed to study the function of *OsSIZ2*. The study revealed positive regulatory influences of *OsSIZ2* on various agronomic traits. Whereas, it′s negative regulatory influences were evident on the concentrations of Pi and total P in different tissues and differential effects on several genes that play pivotal roles in sensing and signaling cascades governing Pi homeostasis. Further, comparative analysis of *ossiz1* and *ossiz2* mutants suggested a partial functional redundancy between *OsSIZ1* and *OsSIZ2*.

## Results

### Expression of *OsSIZ2* is regulated spatiotemporally but not induced during Pi deficiency

**Figure 1:**
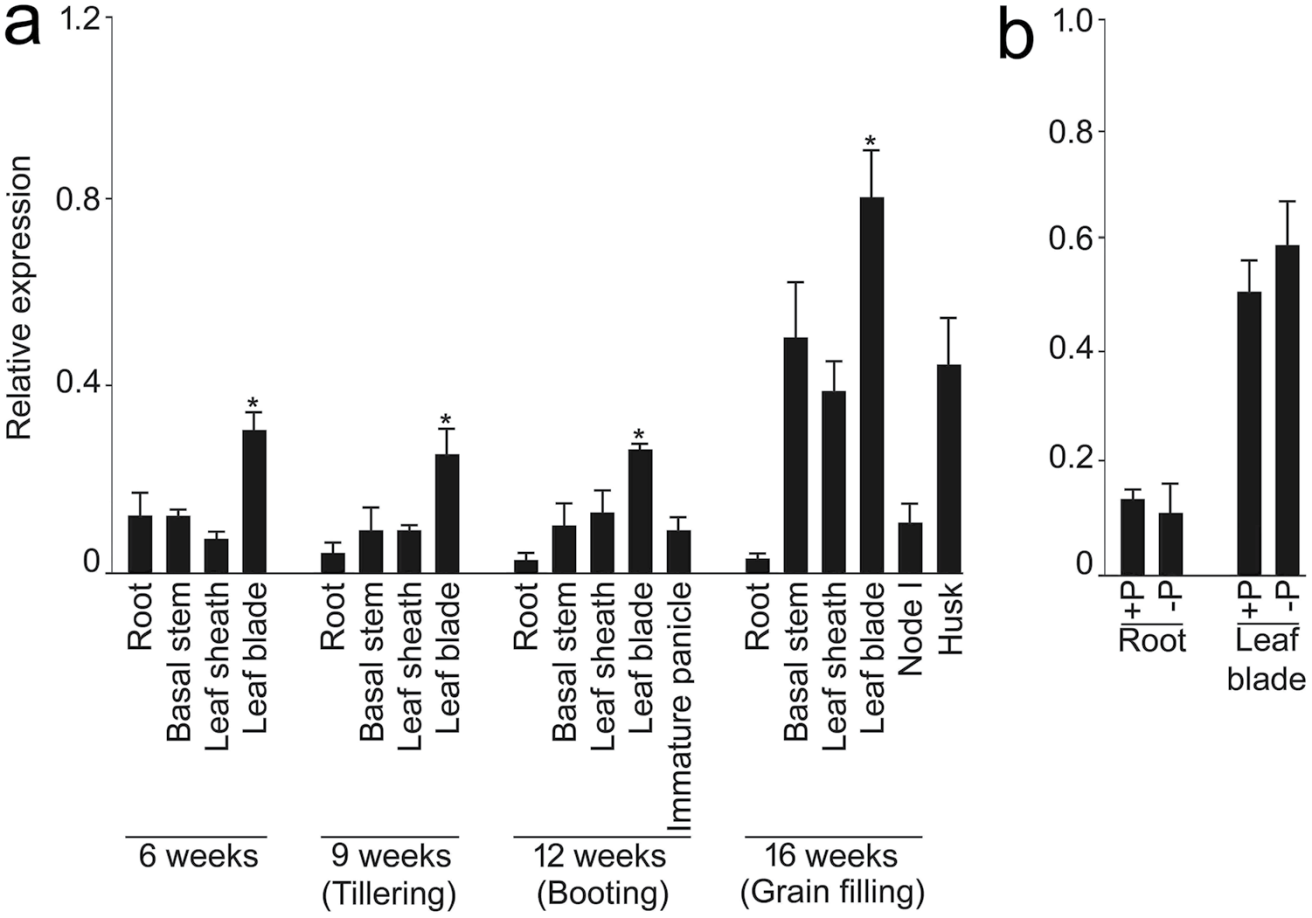
Relative expression levels of *OsSIZ*2 in tissues at different development stages and during growth under different Pi regime. (**a**) Wild-type was grown in a pot soil and tissues were harvested at different time intervals. (**b**) Wild-type seedlings (3-d-old) were grown hydroponically in +P medium (0.3 mM KH_2_PO_4_) for 7 d and then transferred to +P and −P (0 mM KH_2_PO_4_) media for 14 d. The relative expression levels of *OsSIZ2* in the tissues collected from (**a**) and (**b**) were assayed by qRT-PCR. *OsActin* was used as an internal control. Values are means ± SD (*n* = 3). Asterisks indicate that the values were significantly (*P* < 0.05) higher in the leaf blade compared with other tissues.

To determine whether expression of *OsSIZ2* is spatiotemporally regulated, qRT-PCR was employed for assaying its relative expression levels in different tissues collected from 6 to 16-week-old wild-type rice grown in a pot soil (Fig. 1a). Collected samples represented both the vegetative (6 and 9-week-old plants) and reproductive (12 and 16-week-old plants) growth phases. Throughout these growth phases, the relative expression levels of *OsSIZ2* were significantly higher in the leaf blade compared with the other tissues. There were also elevated expression levels of *OsSIZ2* in the basal stem and leaf sheath of a 16-week-old plant compared with the corresponding tissues of 6 to 12-week-old plants. Expression of *OsSIZ2* was also relatively higher in the husk compared with the root and node I at the grain-filling stage. Notably, the expression levels of *OsSIZ2* in the root did not exhibit any significant increment throughout the vegetative and reproductive growth phases. The study revealed the spatiotemporal regulation of *OsSIZ2*. To determine the effects of Pi deficiency on the relative expression levels of *OsSIZ2*, wild-type seedlings were grown hydroponically under different Pi regime for 14 d (Fig. 1b). Pi deficiency did not exert any significant influence on the relative expression levels of this gene in the root and leaf blade.

### Generation of *OsSIZ2* knockout and knockdown mutants for functional characterization

*OsSIZ1*, a paralog of *OsSIZ2*, has been shown to exert regulatory influences on different Pi deficiency responses^22^. Extensive concerted evolution of rice paralogs has led them on to a path of regaining independence^25^. Therefore, elucidation of the level of functional redundancy and divergence in the gene family in rice that plays a pivotal role in the maintenance of Pi homeostasis has often been an attractive paradigm. By employing phylogenetic and mutation analysis, functional redundancy and some degree of functional diversification were observed among four orthologs of *Arabidopsis thaliana* PHOSPHATE RESPONSE1 (PHR1) in rice (OsPHR1-4)^26–28^. Therefore, to determine whether *OsSIZ2* is functionally redundant with *OsSIZ1* and/or exerts distinctive influences on various morphophysiological and molecular responses governing Pi homeostasis, it was functionally characterized. Since reverse genetics is a potent technique for functional genomics in rice^29,30^, it was used for generating T-DNA insertion knockout (*ossiz2* in Dongjin [DJ] background) and RNA interference (RNAi)-mediated knockdown (Ri1-3 in Nipponbare [NB] background) mutants (Supplemental Fig. S1). The insertion of the T-DNA was downstream of the *OsSIZ2* translational start site and in the exon 5 (Supplemental Fig. S1a). Two-round PCR was employed for screening randomly selected 11 putative T-DNA insertion mutants for identifying homozygous *ossiz2* knockout mutants by using (i) T-DNA right border-specific primer P and *OsSIZ2*-specific primer R1 and (ii) *OsSIZ2*-specific primers (F1 and R1) flanking the T-DNA insertion site (Supplemental Fig. S1a,b). The analysis revealed that ~55% of them (lines 3 to 8 marked with red boxes) are homozygous and are hereafter referred to as *ossiz2* (Supplemental Fig. S1b). Semi-quantitative RT-PCR was carried out by using F2 and R2 primers for validating the lack of *OsSIZ2* transcripts in *ossiz2* mutants (lines 3, 5, 7 and 8; Supplemental Fig. S1c). Further, qRT-PCR was employed by using *OsSIZ2*-specific primers for determining the relative expression levels of *OsSIZ2* in wild-types (NB and DJ) and the mutants (Ri1-3 and *ossiz2*) (Supplemental Fig. S1d). The relative expression levels of *OsSIZ2* were significantly reduced (~35–58%) in Ri1-3 and undetected in *ossiz2* compared with their corresponding wild-type. The results confirmed that Ri1-3 and *ossiz2* are knockdown and knockout mutants, respectively of *OsSIZ2*. Southern blot analysis confirmed a single T-DNA insertion in *ossiz2* (Supplemental Fig. S1e).

### Mutation in *OsSIZ2* affects the growth and development

**Figure 2:**
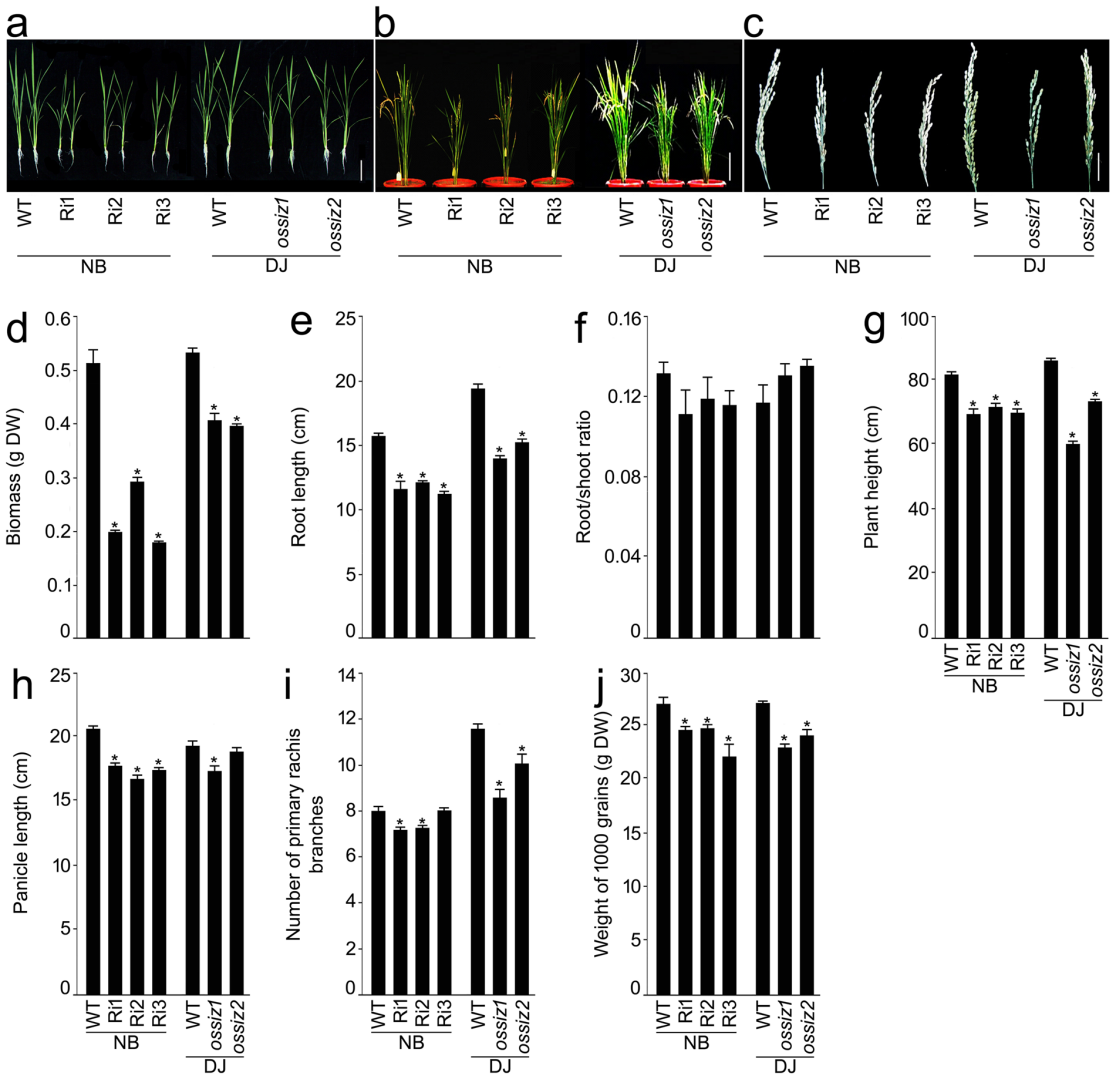
Mutation in *OsSIZ2* affects phenotypic traits at vegetative and reproductive growth phases. (**a**–**c**) Seedlings (3-d-old) of the wild-types (WTs) Nipponbare (NB) and Dongjin (DJ) and RNAi plants (Ri1-3) and T-DNA insertion mutants (*ossiz1* and *2*) in NB and DJ background, respectively were grown (**a**) hydroponically in +P for 4 weeks and (**b**,**c**) in a pot soil for 20 weeks for documenting the phenotypic traits at vegetative (**d**–**f**) and reproductive (**g**–**j**) growth phases, respectively. (**c**) Panicle phenotype of the WTs and the mutants. Bar (**a**–**c**) = 10 cm. Data are presented for the phenotypic traits during vegetative (**d**–**f**; biomass (**d**), root length (**e**) and root/shoot ratio (**f**)) and reproductive (**g**–**j**; plant height (**g**), panicle length (**h**), number of primary rachis branches (**i**) and weight of 1000 grains (**j**)) growth phases. Values are means ± SE (*n* = 10). Asterisks indicate that the values were significantly (*P* < 0.05) lower in the mutants compared with their corresponding WT.

The phenotypic traits of knockdown (Ri1-3) and knockout (*ossiz2*) *OsSIZ2* mutants were compared with their corresponding wild-type and also with *OsSIZ1* mutant (*ossiz1*) during vegetative and reproductive growth phases (Fig. 2). *OsSIZ2* mutant (Ri1-3 and *ossiz2*) seedlings grown hydroponically in a +P medium for 4 weeks revealed retarded vegetative growth compared with their corresponding wild-type and their responses were analogous to *ossiz1* (Fig. 2a). Similar attenuating effects were also explicit on the growth responses of these mutants (Ri1-3, *ossiz1* and *ossiz2*) compared with their respective wild-type when grown to maturity (20-week-old) in a pot soil (Fig. 2b). The inhibitory effects of the mutation in *OsSIZ1* and *OsSIZ2* were also apparent in the development of the panicle (Fig. 2c). Further, effects of the mutation in *OsSIZ1* and *OsSIZ2* on various morphometric traits were compared with their respective wild-type (Fig. 2d–j). Ri1-3 showed significant reductions in biomass, root length, plant height, panicle length and weight of 1000 grains compared with NB (Fig. 2d,e,g,h and j). The number of primary rachis branches was significantly lower in Ri1 and 2 (but not in Ri3) compared with NB (Fig. 2i). However, root/shoot ratio of Ri1-3 was comparable with NB (Fig. 2f). The *ossiz1* and *ossiz2* mutants also exhibited the reductions in biomass, root length, plant height, the number of primary rachis branches and weight of 1000 grains compared with DJ (Fig. 2d,e,g,i and j). Compared with *OsSIZ2*, the effects of the mutation in *OsSIZ1* were relatively more aggravated on some of these morphometric traits (Fig. 2e,g,i and j). This trend was more explicit with respect to panicle length, which was significantly lower in *ossiz1* compared with DJ and *ossiz2* (Fig. 2h). The present study thus suggested a pivotal role of *OsSIZ2* in exerting positive regulatory influences on the developmental responses of different morphometric traits during vegetative and reproductive growth phases. Comparative analyses of these traits in *ossiz2* with *ossiz1* also revealed some degree of functional redundancy between *OsSIZ2* and *OsSIZ1*.

### Mutation in *OsSIZ2* affects uptake and mobilization of Pi during vegetative growth

**Figure 3:**
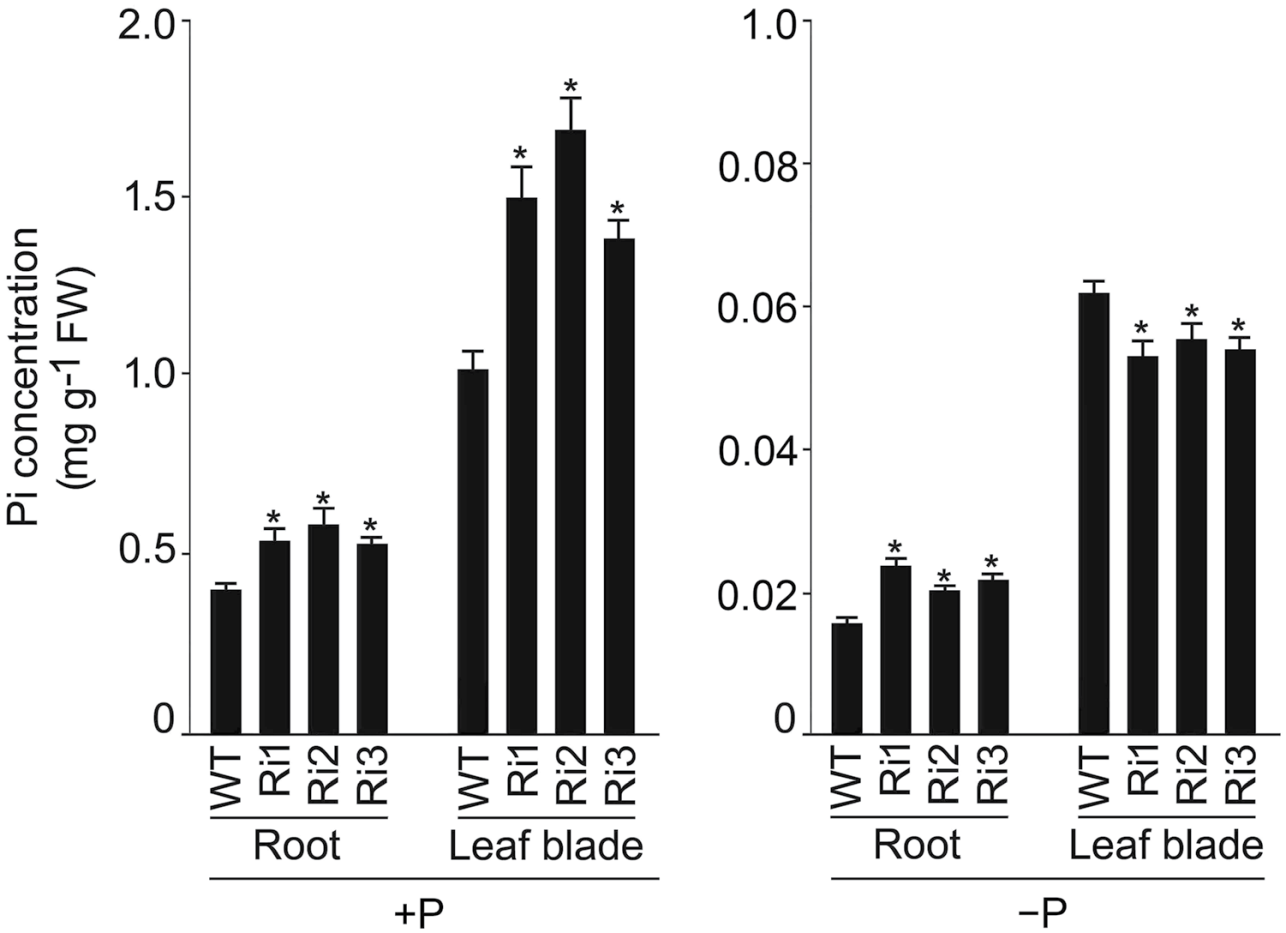
RNAi-mediated silencing of *OsSIZ2* affects Pi concentration under different Pi regimes. Seedlings (3-d-old) of NB and RNAi plants (Ri1-3) in NB background were grown hydroponically in +P and −P media for three weeks and their root and leaf blade were assayed for Pi concentration. Values are means ± SE (*n* = 4) and asterisks indicate that the values were significantly (*P* < 0.05) different in the mutants compared with the WT. FW, Fresh weight.

**Figure 4:**
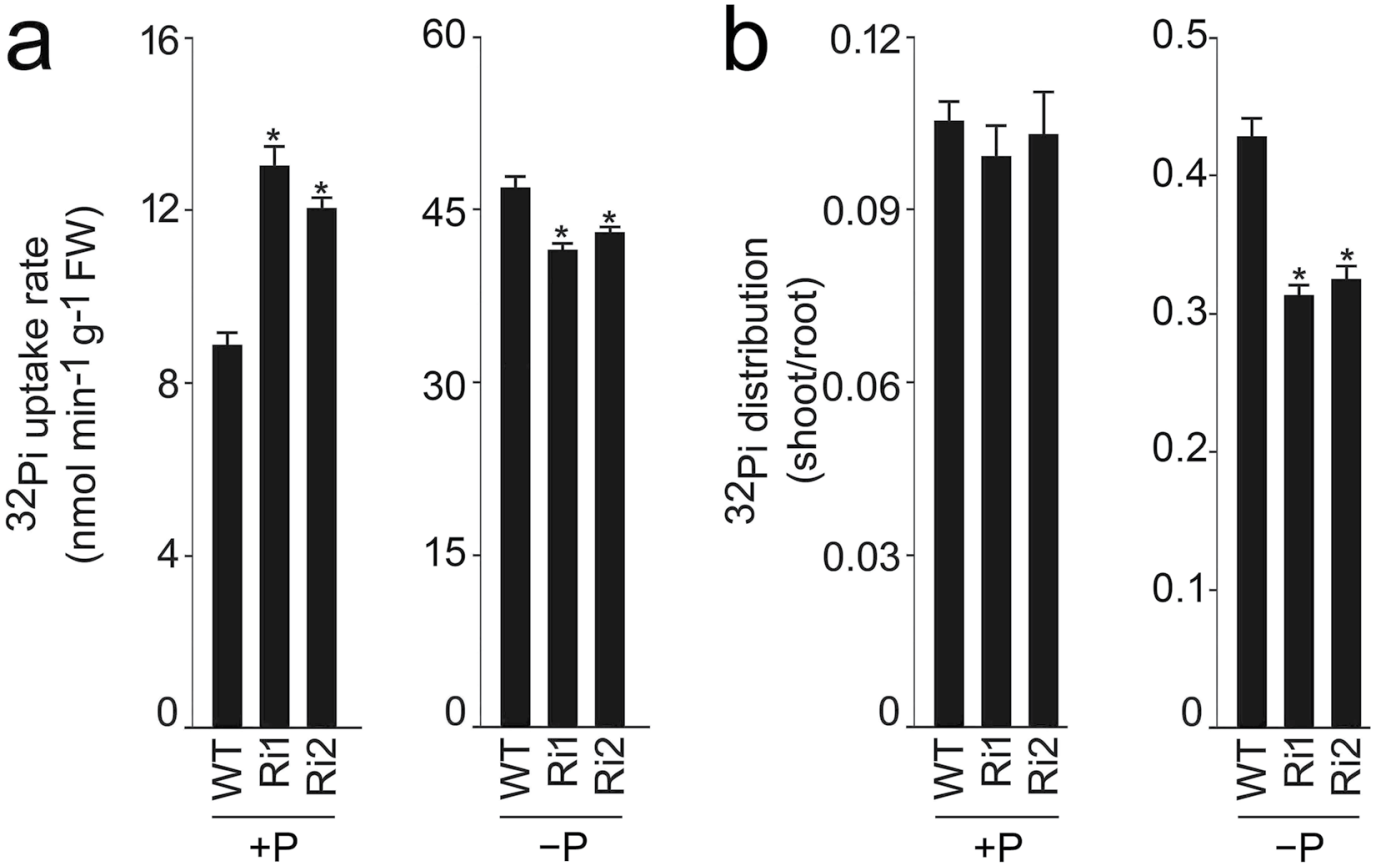
RNAi-mediated silencing of *OsSIZ2* affects uptake rate and distribution of ^32^Pi under different Pi regime. Rice seedlings (10-d-old) of NB and Ri1-2 were grown hydroponically in +P and −P media for 7 d. Seedlings were then transferred to +P and −P media labeled with ^32^Pi for 3 h. Roots and shoots of the labeled seedlings were separated. (**a**) Root was assayed for ^32^Pi uptake rate. (**b**) Root and shoot were assayed to determine the distribution of ^32^Pi between them. Values are means ± SE (*n* = 4) and asterisks indicate that the values were significantly (*P* < 0.05) different in the mutants compared with the WT.

**Figure 5:**
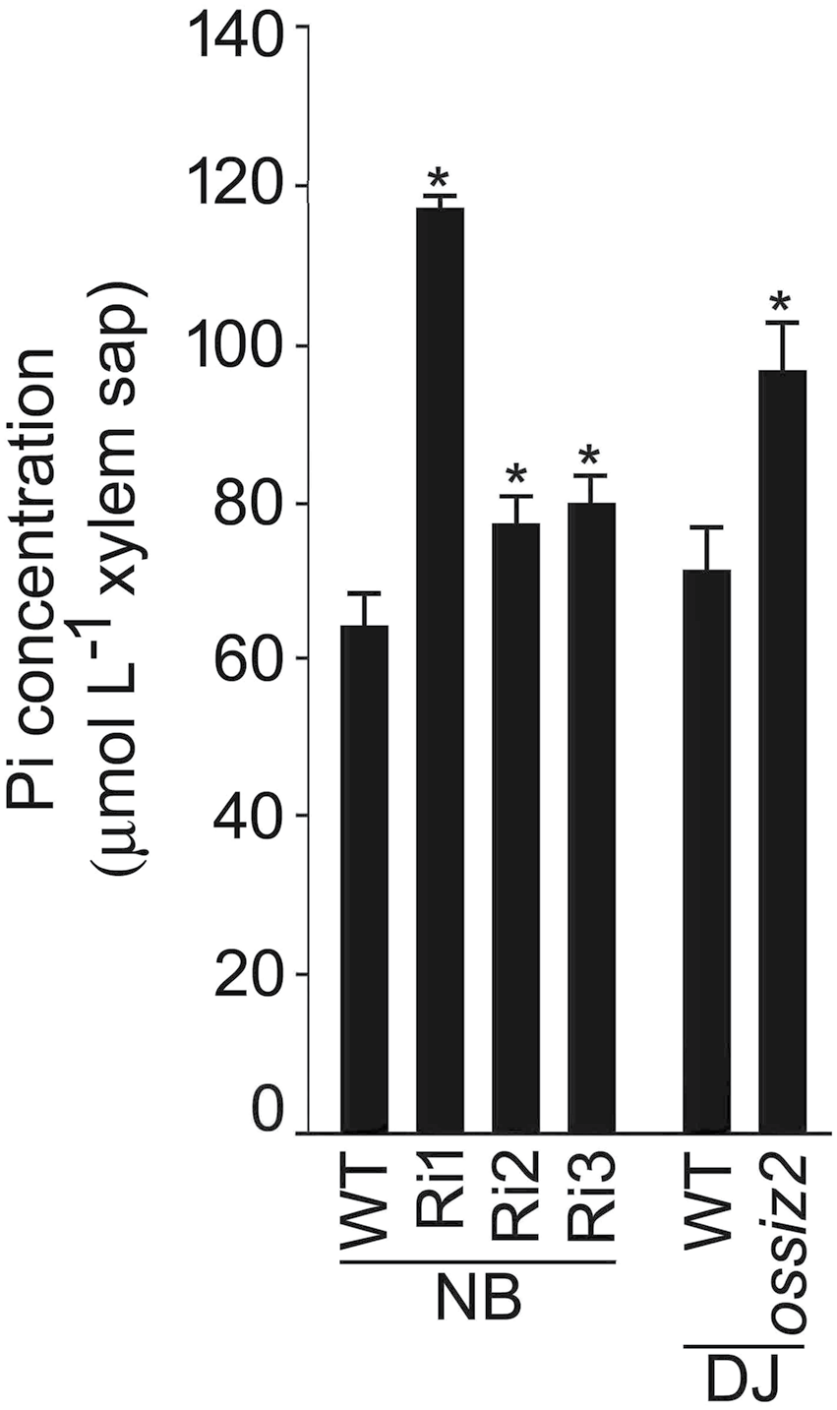
Pi concentration in the xylem sap is elevated in *OsSIZ2* mutants. WTs (NB and DJ) and the mutants (Ri1-3 and *ossiz2*) were grown in a pot soil for 16 weeks (grain-filling stage). Xylem sap was collected and assayed for Pi concentration. Values are means ± SE (*n* = 5) and asterisks indicate that the values were significantly (*P* < 0.05) higher in the mutants compared with their corresponding WT.

Since *OsSIZ1* has been shown to play a role in the maintenance of Pi homeostasis^22^, it raised a pertinent question about a likely involvement of *OsSIZ2*. To investigate this, seedlings (3-d-old) of the wild-type (NB) and Ri1-3 were grown hydroponically for three weeks in +P and −P media and their root and leaf blade were assayed for Pi concentration (Fig. 3). Under +P condition, there were significant increases in the concentration of Pi in the root (~34–48%) and leaf blade (~36–65%) of Ri1-3 compared with the wild-type. Under −P condition, the concentration of Pi significantly increased in the root (~29–51%) and decreased in the leaf blade (~10–15%) of Ri1-3 compared with the wild-type. It was assumed that higher Pi concentration in the root (+P and −P) and leaf blade (+P) of Ri1-3 mutants could be due to elevated Pi acquisition by their roots. To investigate this, uptake rate (root) and distribution (shoot/root) of ^32^Pi was compared between the wild-type (NB) and the mutants (Ri1 and 2) under +P and −P conditions (Fig. 4). Although under +P condition, there was significantly higher (~36–47%) ^32^Pi uptake rate by roots of Ri1 and 2 compared with the wild-type, the corresponding values were significantly lower (~8–11%) under −P condition (Fig. 4a). The effects of different Pi regime were also evident on the ^32^Pi distribution (shoot/root) with the value being comparable between the wild-type and the mutants under +P condition, while it was significantly lower (~24–27%) in the mutants compared with the wild-type under −P condition (Fig. 4b). In an earlier study, the efficacy of Pi mobilization from the root to shoot was positively correlated with the Pi concentration in xylem sap^31^. Therefore, Pi concentration in the xylem sap was assayed in the culms of wild-types (NB and DJ) and the mutants (Ri1-3 and *ossiz2*) grown in a pot soil up to the grain-filling stage (Fig. 5). Pi concentration in the xylem sap was significantly higher in Ri1-3 (~21–83%) and *ossiz2* (~35%) compared with their corresponding wild-type.

### Mutation in *OsSIZ2* triggered elevated total P concentration in different tissues at grain-harvest stage

**Figure 6:**
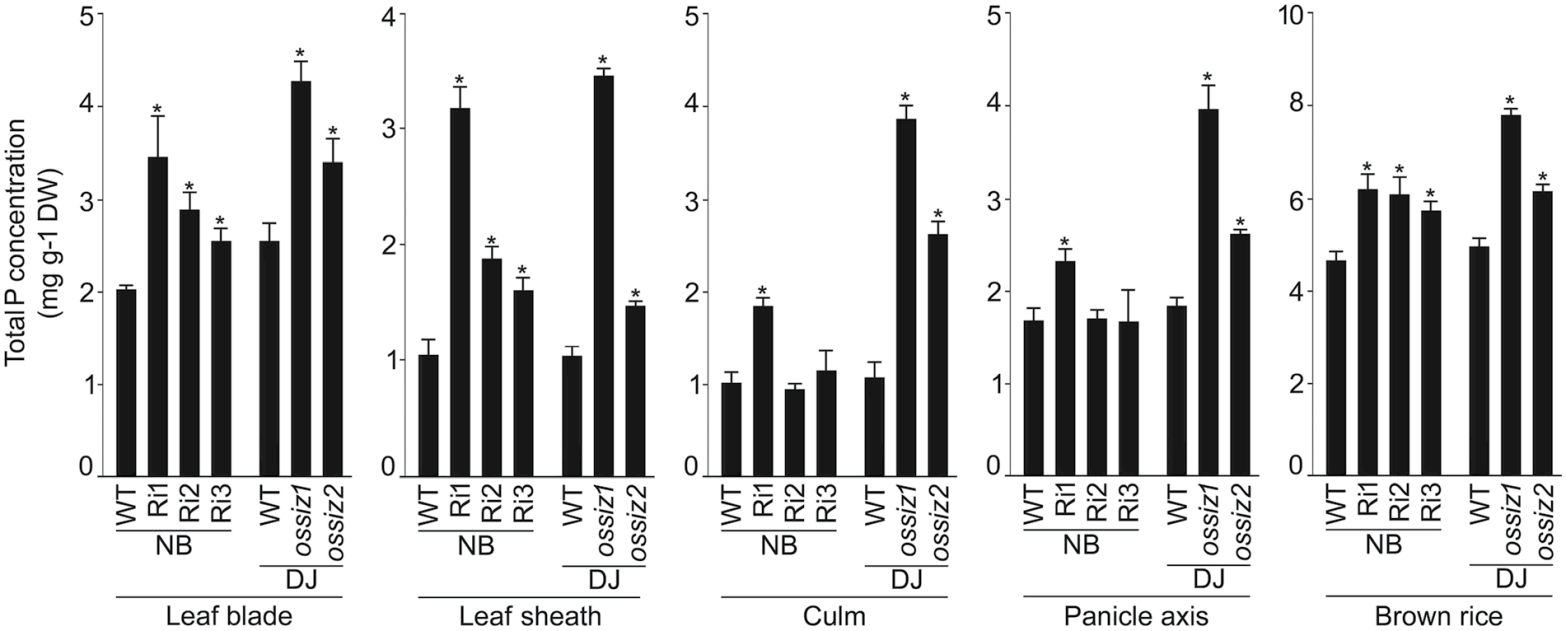
Mutation in *OsSIZ2* triggers accentuated total P concentration. WTs (NB and DJ) and the mutants (Ri1-3, *ossiz1* and 2) were grown in a pot soil for 20 weeks (grain-harvest stage). Data are presented for total P concentration in different tissues. Values are means ± SE (*n* = 5) and asterisks indicate that the values were significantly (*P* < 0.05) higher in the mutants compared with their corresponding WT. DW, Dry weight.

Total P concentration was assayed in different tissues of the wild-types (NB and DJ) and the mutants (Ri1-3, *ossiz1* and *ossiz2*) grown in a pot soil up to the grain-harvest stage (Fig. 6). Total P concentration was significantly higher in Ri1 (~72% in leaf blade, ~3-fold in leaf sheath, ~44% in culm, ~27% in panicle axis and ~24% in brown rice) compared with NB. Total P concentration of Ri2 and 3 in leaf blade, leaf sheath and brown rice was relatively lower than Ri1, but significantly higher compared with NB. Whereas, total P concentration of Ri2 and 3 in culm and panicle axis was comparable with NB. The data suggested that among the three *OsSIZ2*-RNAi transgenic lines, Ri1 was more robust in augmenting the total P concentration in different tissues. This accentuated response of Ri1 was consistent with its higher ^32^Pi uptake rate and Pi concentration in the xylem compared with Ri2 and/or 3 under +P condition (Figs 4a and 5). Although a similar trend of significantly elevated total P concentration was also observed in these tissues of *ossiz1* and *ossiz2* compared with DJ, the effect was more pronounced in *ossiz1* than *ossiz2*.

### Mutation in *OsSIZ2* differentially affects the relative expression levels of a subset of genes involved in the maintenance of Pi homeostasis

**Figure 7:**
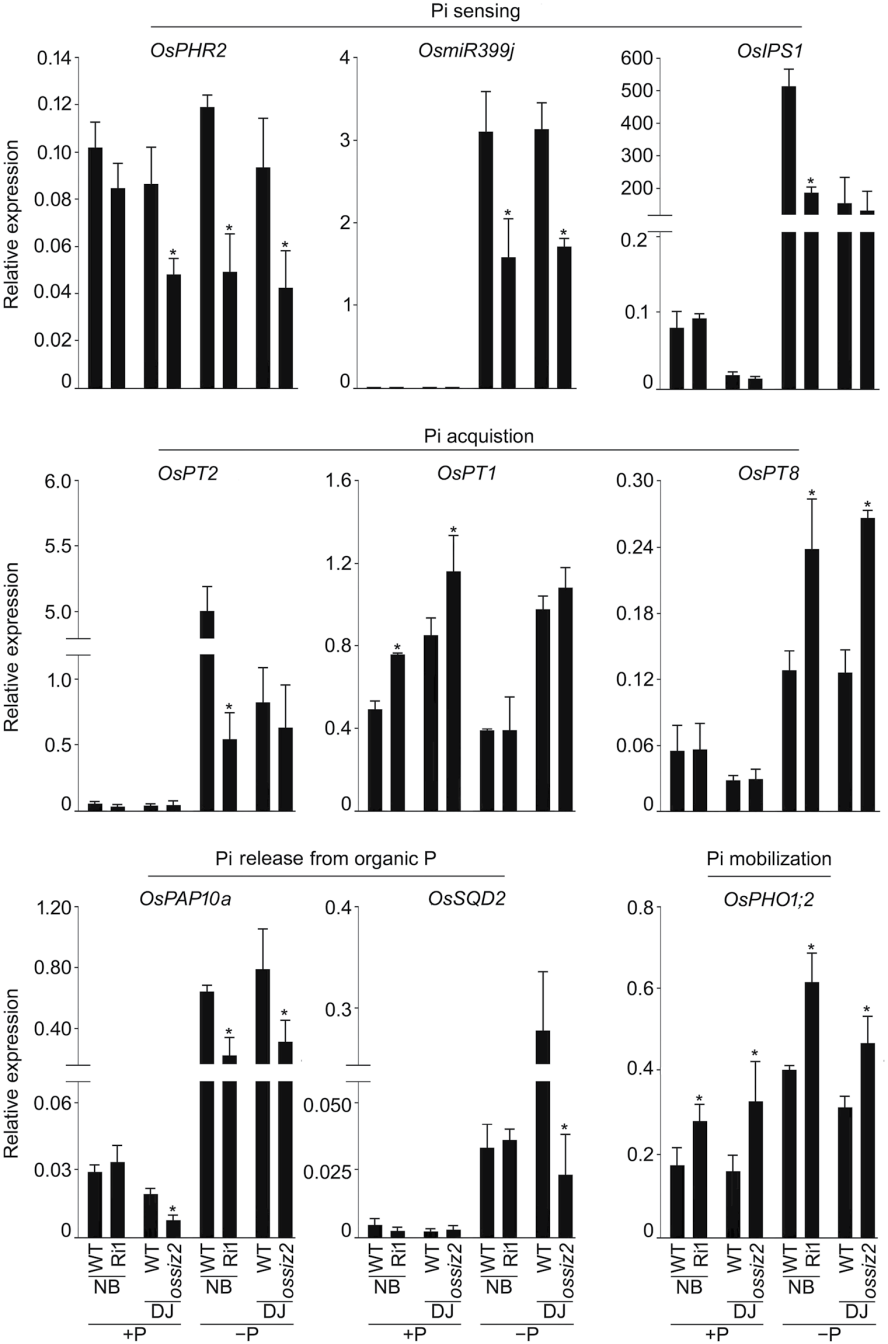
Mutation in *OsSIZ*2 exerts differential effects on the relative expression levels of the genes involved in Pi homeostasis. WTs (NB and DJ) and the mutants (Ri1 and *ossiz2*) were grown hydroponically under +P and −P conditions as described in the legend to Fig. 1b. Root tissues were subjected to qRT-PCR analysis for determining the relative expression levels of functionally different genes implicated in the maintenance of Pi homeostasis. *OsActin* was used as an internal control. Values are means ± SD (*n* = 3). Asterisks indicate that the values differ significantly (*P* < 0.05) in the mutants compared with their corresponding WT.

Several genes involved in the sensing and signaling cascades that govern Pi homeostasis have been functionally characterized in rice^32^. Therefore, qRT-PCR was employed for assaying the relative expression levels of some of these genes in the roots of the wild-types (NB and DJ) and the mutants (Ri1 and *ossiz2*) grown hydroponically under +P and −P conditions for 14 d (Fig. 7). Biomass and root/shoot ratio of the mutants (Ri1-3) and were significantly (*P* < 0.05) attenuated compared with the wild-type under −P condition (data not shown). Under +P condition, the relative expression levels of *OsPT1* and *OsPHO1*;*2* in Ri1 and *ossiz2* were significantly higher, while that of *OsPHR2* and *OsPAP10a* were attenuated in *ossiz2* compared with their respective wild-type. Under −P condition, the relative expression levels of these genes varied in the mutants ranging from suppression (*OsPHR2*, *OsmiR399j* and *OsPAP10a*) and induction (*OsPT8* and *OsPHO1*;*2*) in both Ri1 and *ossiz2*, suppression only in Ri1 (*OsIPS1* and *OsPT2*) and *ossiz2* (*OsSQD2*) and no effect on either of these mutants (*OsPT1*) compared with their corresponding wild-type. Overall, the relative expression analysis of Ri1-3 and *ossiz2* provided evidence towards the differential regulatory influence of *OsSIZ2* on a subset of molecular entities that governs Pi homeostasis in rice.

### *OsSIZ1* exerts regulatory influence on *OsSIZ1* in roots

**Figure 8:**
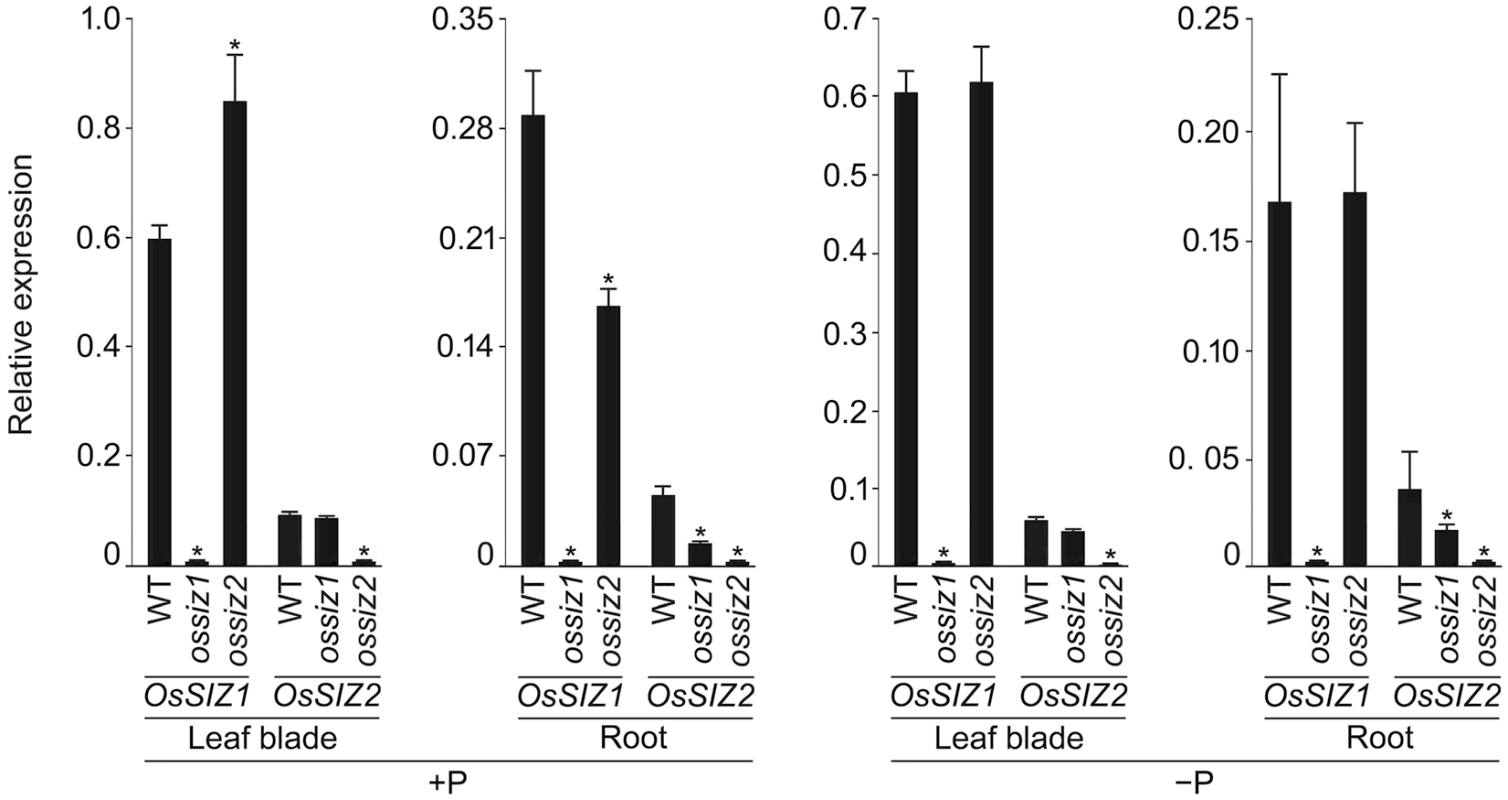
Relative expression levels of *OsSIZ1* and *OsSIZ2* in leaf blade and root of *ossiz1* and *ossiz2* under different Pi regime. WT (DJ) and the mutants (*ossiz1* and *2*) were grown hydroponically under +P and −P conditions as described in the legend to Fig. 1b. qRT-PCR was employed for determining the relative expression levels of *OsSIZ1* and *OsSIZ2* in the leaf blade and root of these seedlings. *OsActin* was used as an internal control. Values are means ± SD (*n* = 3). Asterisks indicate that the values were significantly (*P* < 0.05) different in the mutants compared with the WT.

The relative expression levels of *OsSIZ1* and *OsSIZ2* were assayed in the leaf blade and root of the wild-type, *ossiz1* and *ossiz2* seedlings grown hydroponically under +P and −P conditions for 14 d (Fig. 8). As anticipated, there were barely any detectable expression levels of *OsSIZ1* and *OsSIZ2* in the leaf blade and root of *ossiz1* and *ossiz2*, respectively under different Pi regime. In *ossiz2*, the relative expression levels of *OsSIZ1* were higher in +P leaf blade, lower in +P root and were unaffected in Pi-deprived leaf blade and root of *ossiz2* as compared with the wild-type. Whereas in *ossiz1*, the relative expression levels of *OsSIZ2* were attenuated in root and remained unaffected in leaf blade as compared with the wild-type under different Pi regime. The result provided some evidences towards the regulatory influence of *OsSIZ1* on *OsSIZ2* in roots of the seedlings grown under different Pi regime.

## Discussion

Post-translational modifications (PTMs) are critical in determining the activity of proteins, which then mediate complex regulatory processes that are important for the functioning of a cell. These PTMs include processes such as phosphorylation, sumoylation, ubiquitination, N-terminal acetylation, carbonylation etc^33^. Of these, sumoylation conjugates Small Ubiquitin-related MOdifier (SUMO) to substrate proteins through reversible post-translational modifications^34^. It is implicated in transcriptional activation, degradation and localization of proteins, protein-protein interactions and is highly regulated by the environment^35^. SUMO conjugation (Sumoylation) to the target proteins involves the sequential actions of activation (E1), conjugation (E2) and ligation (E3)^36^. There is one member of the Protein Inhibitor of Activated STAT (PIAS) group of SUMO E3 ligases (SIZs) in Arabidopsis (*AtSIZ1*) and two members in rice (*OsSIZ1*and *OsSIZ2*)^17^.

*OsSIZ2* showed ubiquitous expression in different vegetative (6 to 9-week-old) and reproductive (12 to 16-week-old) tissues with a relatively higher level in the leaf blade during growth in a pot soil (Fig. 1a). Further, there were increments in the expression of *OsSIZ2* in different tissues as the development progressed from the vegetative to reproductive phase of the life cycle (Fig. 1a). This suggested potential roles of *OsSIZ2* in growth and development throughout the life cycle in rice. *OsSIZ1* also showed constitutive expression in different tissues with the expression being strongest in the leaves^22^. OsSIZ1 and OsSIZ2 exhibit SUMO E3 ligase activity and are localized to the nucleus^20^.

Reverse genetic approaches of T-DNA insertion-mediated knockout and/or RNA interference (RNAi)-mediated knockdown has expedited the process of functional genomics of rice, which is the first sequenced crop genome^37–39^. The reverse genetic approach was used for the functional characterization of *OsSIZ1* ^22,24^. Therefore, to functionally characterize the role of *OsSIZ2*, knockout (*ossiz2*) and knockdown (Ri1-3) mutants were generated (Supplemental Fig. S1). The locations of T-DNA insertions are preferred in the order of exon, intron, 5′-untranslated region and the promoter for effectively knocking out the function of the gene^40^. Consistent with this notion, the insertion of the T-DNA was found to be in the exon 5 of *OsSIZ2* (Supplemental Fig. S1a), which suggested its potential efficacy of knocking out the function of this gene. This was confirmed by qRT-PCR analysis, which revealed a lack of any transcripts of *OsSIZ2* in *ossiz2* mutant (Supplemental Fig. S1d). This was consistent with a review of about 1000 research published papers, which revealed that T-DNA insertion in exon was effective in generating a gene knock out ~90% of the time^41^. Further, Southern blot analysis validated a single T-DNA insertion in *ossiz2* (Supplemental Fig. S1e). In addition, the expression of *OsSIZ2* was significantly reduced (~35–58%) in Ri1-3 (Supplemental Fig. S1d). The mutants (*ossiz2* and Ri1-3) thus provided valuable genetic tools to determine the function of *OsSIZ2*.

To determine the effects of the mutation in *OsSIZ2* on vegetative growth phase, seedling (3-d-old) of the wild-types (NB and DJ) and the mutants (Ri1-3 and *ossiz2*) were grown hydroponically in +P medium for 4 weeks (Fig. 2a,d–f). There were significant reductions in the biomass and root length of the mutants compared with their respective wild-type. This suggested a positive regulatory influence of *OsSIZ2* on these vegetative traits. An earlier study also showed attenuation in primary root growth of *ossiz1* (3-week-old) seedlings grown in hydroponic culture^24^. Auxin plays a pivotal role in root morphogenesis and development^42,43^. In Arabidopsis, *SIZ1* has been implicated in the remodelling of root architecture by regulating auxin patterning^19^. Genetics and functional genomics have expedited in the identification of several genes that play key roles in controlling root development in rice^44^. It would be interesting to know if any of these genes involved in root development could potentially be sumoylated by *OsSIZ1* and/or *OsSIZ2* and thus warrants further detailed studies. To further determine the effects of the mutation in *OsSIZ2* on any of the reproductive traits, wild-types and the mutants (*ossiz2* and Ri1-3) were grown in a pot soil for 20 weeks (Fig. 2b,c,g–j). These mutants showed significant reductions in plant height, the number of primary rachis branches and weight of 1000 grains compared with the wild-types. This clearly suggested broad-spectrum positive regulatory influences of *OsSIZ2* on growth and development during vegetative and reproductive growth phases. *OsSIZ1* has been also been implicated in regulating several traits representing reproductive growth phase^23,24^. Since traits governing growth and development are polygenic, identifying sumoylated proteins *in vivo* can be a daunting task. In this context, an integrative analysis of known and putative SUMO substrates could provide a rich repository of SUMO-regulated events^45^. Although studies on mining SUMO targets revealed that majority of them were transcription factors and genes involved in chromatin-related processes and/or RNA/DNA-dependent processes, some of them were not nucleus-localized^46^. A novel proteomic approach involving 2-D liquid chromatography, immunodetection and mass spectroscopy analyses have also been demonstrated for the identification of novel SUMO targets^47^. Overall, these integrative approaches can provide valuable insights into the mechanistic details of the sumoylated-mediated regulation of growth and development in rice^48^.

Now there are growing evidence to implicate sumoylation in the maintenance of nutrient homeostasis. For instance in Arabidopsis, sumoylation plays key roles in Pi starvation responses^18^, tolerance to excess copper^49^ and stimulation of nitrate reductase activity^50^. Recently in rice, *OsSIZ1* has also been shown to exert regulatory influences on several traits governing homeostasis of Pi and N^22^. *OsSIZ2* was not induced in Pi-deprived root and leaf blade (Fig. 1b). Non-responsiveness of *OsSIZ2* to Pi deficiency was consistent with that of *OsSIZ1* ^22^. This suggested that sumoylation may be responding to different Pi regime by modification of proteins involved in the maintenance of Pi homeostasis downstream of *OsSIZ1* and/or *OsSIZ2*. Furthermore, several genes that play a key role in the maintenance of Pi homeostasis such as transcription factor *OsPHR1* and *OsPHR2* ^26^, Pi transporter *OsPT1* ^51^ and *OsSPX4* ^52^ show constitutive expression irrespective of Pi regime. Elevated concentrations of Pi and total P in different tissues, and higher ^32^Pi uptake rate in the mutants (Ri1-3 and/or *ossiz2*) under +P condition (Figs 3–6) suggested negative regulatory influences of *OsSIZ2* on physiological traits governing Pi homeostasis. A similar negative regulatory influence of *OsSIZ2* was also evident on the relative expression levels of *OsPT1* under +P condition, *OsPT8* under −P condition and *OsPHO1*;*2* under both +P and −P conditions (Fig. 7). On the contrary, *OsSIZ2* exerted positive regulatory influences on the uptake and distribution of ^32^Pi under −P condition and the relative expression levels of *OsPHR2*, *OsmiR399a*, *OsIPS1*, *OsPT2*, *OSPAP10* and *OsSQD2* (Figs 4 and 7). This suggested that *OsSIZ2* acts both positively and negatively on different molecular entities that govern Pi homeostasis. *OsSIZ1* and *SIZ1* in Arabidopsis also exhibited differential regulatory influences on the genes involved in Pi homeostasis^18,22^. Attenuation in the relative expression of *OsSIZ1* in +P root of *ossiz2* and that of *OsSIZ2* in −P root of *ossiz1* (Fig. 8) suggested reciprocal influences on each other. Interestingly, a comparative analysis of the effects of the mutation on *OsSIZ1* ^22^ and *OsSIZ2* (Fig. 7) on the relative expression levels of Pi transporters in the roots of *ossiz1* and *ossiz2* mutants revealed their positive (*OsPT2*) and negative (*OsPT1* and *OsPT8*) regulatory influences. This suggested a prevalence of functional redundancy between these two paralogs. It is not surprising because members of the gene families in rice genome have a high probability (~51%) of being functionally redundant^53,54^. But notably, the negative regulatory influence of *OsSIZ1* on *OsPT1* was under −P condition^22^, whereas that of *OsSIZ2* on this Pi transporter was under +P condition (Fig. 7). This indicated at least some degree of functional independence between these two genes.

Further, to get a better insight into the relative effects of the mutation in *OsSIZ1* and *OsSIZ2* on different agronomic traits, the wild-type (DJ) and the mutants (*ossiz1* and *ossiz2*) were grown hydroponically in +P medium (4 weeks) and in a pot soil (20 weeks) for documenting the effects on the vegetative and reproductive traits (Fig. 2). Although during vegetative growth phase the reduction in biomass in both these mutants was comparable, the attenuating effect on root length was more robust in *ossiz1* compared with *ossiz2* (Fig. 2a,d,e). Relatively more aggravated effects of *ossiz1* on the reproductive traits compared with *ossiz2* were also evident (Fig. 2b,c,g–j). The results suggested a relatively dominant positive regulatory effect of *OsSIZ1* compared with *OsSIZ2* during vegetative and reproductive growth phases of rice. An earlier study has also shown that the expression of *OsSIZ1* was ~2-fold higher than *OsSIZ2* in different vegetative and reproductive tissues^55^. Similarly, the negative regulatory effects of *OsSIZ1* were more pronounced than *OsSIZ2* on the total P concentration in different tissues (Fig. 6). Together the results suggested dominant regulatory effects of *OsSIZ1* compared with *OsSIZ2* on the responses related to growth and development and maintenance of Pi homeostasis.

## Materials and Methods

### Plant materials and growth conditions

Rice (*Oryza sativa* L. ssp *japonica*) wild-type (Nipponbare [NB] and Dongjin [DJ]), T-DNA insertion mutants (*ossiz1* and *2*) and *OsSIZ2* RNAi plants (Ri1-3) in DJ and NB background, respectively were used in this study. Seeds were surface-sterilized as described^22^ and germinated on half-strength Murashige and Skoog (MS) medium in dark at 25 °C for 3 d. Seedlings with their radicle length in the range of 2–3 cm were transferred to a hydroponic set up containing +P medium and maintained in a growth room (16-h-light [30 °C]/8-h-dark [22 °C] and relative humidity at ~70%) for 7 d. +P medium comprised KH_2_PO_4_ (0.3 mM), NH_4_NO_3_ (1.25 mM), CaCl_2_ (1 mM), MgSO_4_ (1 mM), Na_2_SiO_3_ (0.5 mM), K_2_SO_4_ (0.35 mM), Fe-EDTA (20 µM), H_3_BO_3_ (20 µM), MnCl_2_ (9 µM), ZnSO_4_ (0.77 µM), Na_2_MoO_4_ (0.39 µM) and CuSO_4_ (0.32 µM) with pH adjusted to 5.5. Seedlings were then transferred to a hydroponic set up containing +P and −P (0 mM KH_2_PO_4_) media for 14 d. The nutrient media in the hydroponic set up were replaced every 3 d during the course of the experiment. Soil collected from an experimental farm at Nanjing Agricultural University was used for the pot experiments as described^22^.

### Identification of *ossiz2* mutant

*OsSIZ2* mutant line PFG_3A-13223.R with T-DNA from pGA2715 vector in DJ background was obtained from RiceGE (http://signal.salk.edu/RiceGE). Two-round PCR was performed for screening the homozygous mutants. The first-round PCR was carried out with T-DNA-specific primer (P) and *OsSIZ2*-specific primers (R1) for identifying the mutants with T-DNA insertion. Subsequently, second-round PCR was performed with the primers (F1 and R1) flanking the T-DNA insertion site for identifying homozygous mutants. *OsActin* was used as the reference gene. A list of primers used is given in Supplementary Table S1.

### Generation of *OsSIZ2*-RNAi transgenics

To generate RNAi construct, *OsSIZ2* coding sequence-specific 479 bp fragment (1050–1529 bp downstream of the ATG start codon defined as 1) was amplified by PCR. To facilitate subsequent cloning of the PCR product into the binary vector pTCK303, *Bam*HI and *Kpn*I sites were incorporated into the 5′ end of both forward primer AS-F (5′-GGATCCTTAAGACGGCCACCTGTTTC-3′) and reverse primer AS-R (5′-GTGGTACCGAGGCAGATAATGCTGACAG-3′). The purified PCR product was ligated to the TA cloning pMD18-T vector (TaKaRa) and positive clones were selected. The PCR-amplified fragment and pTCK303 were digested with *Bam*HI and *Kpn*I and cloned in the sense orientation as described^56^. *OsSIZ2*-specific 479 bp fragment was then amplified using forward primer S-F (5′-ATTGAGCTCTTAAGACGGCCACCTGTTTC-3′) and reverse primer S-R (5′-GGTACTAGTGAGGCAGATAATGCTGACAG-3′) with *Sac*I and *Spe*I sites incorporated into their 5′ end. The PCR-amplified fragment was cloned in the antisense orientation in pTCK303 using a similar strategy. The presence of the two inserts in the desired orientation in the plasmid was confirmed by sequencing. The plasmid was transferred to *Agrobacterium tumefaciens* strain EHA105 by electroporation and then transformed into embryonic calli (induced on N6 medium) of NB as described^57^. Transgenic plants were selected on a medium containing hygromycin (50 mg L^−1^), transferred to the soil and grown to maturity. Three independently generated *OsSIZ2*-RNAi transgenic lines (Ri1-Ri3) were selected. qRT-PCR was used for validating the knockdown of the relative expression levels of *OsSIZ2* in Ri1-Ri3 compared with the wild-type (Supplemental Fig. S1d).

### Semiquantitative RT-PCR and qRT-PCR analyses

Total RNAs from various tissues of the wild-types (NB and DJ) and the mutants (*ossiz1* and *2* and Ri1-3) was isolated using Trizol reagent (Invitrogen). Semi-quantitative RT-PCR was carried out by using gene-specific primers for *OsSIZ2*. The PCR products were analyzed on agarose gel (1%, w/v) and images were captured with a CCD camera. For qRT-PCR, total RNA (~2 µg) was treated with RNase-free DNase I and reverse transcribed using SuperScript III first-strand synthesis kit (Invitrogen). qRT-PCR analysis was performed on a StepOnePlus Real-Time PCR System (Applied Biosystem) using SYBR green master mix (Vazyme) and gene-specific primers. The relative expression levels of the genes were computed by 2^–ΔΔ*C*T^ method of relative quantification^58^. *OsActin* was used as a control for both semiquantitative RT-PCR and qRT-PCR analyses. A list of primers used is given in Supplementary Table S1.

### Southern blot analysis

The copy number in *ossiz2* was determined by Southern blot analysis. The genomic DNA (80–100 μg) was digested overnight with *Hind*III and *Bgl*II at 37 °C, separated on agarose gel (1%, w/v) and blotted onto a nylon membrane (Hybond N+, Amersham). The membrane was hybridized overnight in a hybridization solution as described^59^ containing DIG-labeled *hygromycin phosphotransferase* (25 ng ml^−1^) probe. The membrane was washed twice with a solution containing 1xSSC and SDS (0.1%, w/v) for 15 min at 65 °C. The washed membrane was analyzed with ScanMaker S260 (Microtek).

### Quantification of Pi

Wild-type (NB) and the mutants (Ri1-3) were grown hydroponically in +P and −P media for three weeks and their root and leaf blade were harvested. For collection of the xylem sap, the wild-types (NB and DJ) and the mutants (Ri1-3 and *ossiz2*) were grown in a pot soil for 16 weeks (grain-filling stage). An incision was made on their stem at 4 cm above the soil surface. The cut surface was rinsed with deionized water and blotted dry. A cotton wool pad was placed on the cut surface for 12 h to facilitate the absorption of xylem sap. Pi concentration in the tissues (root and leaf blade) and xylem sap (extracted from the cotton wool) was assayed as described^60^.

### Quantification of total P

Wild-types (NB and DJ) and the mutants (Ri1-3, *ossiz1* and *2*) were grown in a pot soil for 20 weeks (grain-harvest stage) and different tissues (leaf blade, leaf sheath, culm, panicle axis and brown rice) were harvested. Dry tissues (~50 mg) were digested with H_2_SO_4_ (5 ml) in glass tubes overnight at room temperature. The tubes were then heated to 280 °C and H_2_O_2_ (6–8 drops) were added at an interval of 10 min until the solution turned colourless. The digested sample was diluted to 100 ml with deionized water and total P concentration was assayed as described^60^.

### Assay for the uptake and distribution of ^32^Pi

Rice seedlings (10-d-old) of the wild-type (NB) and the mutants (Ri1,2) were grown in +P and −P media for 7 d. The seedlings were then transferred to the nutrient solution supplemented with ^32^Pi (8 μCi, Perkin-Elmer) and grown for 3 h. To remove the apoplastic ^32^Pi, roots were washed thrice for 5 min each with ice-cold desorption solution (2 mM MES, 0.5 mM CaCl_2_ and 0.1 mM NaH_2_PO_4_, pH 5.5). Seedlings were blotted-dry, root and shoot were separated and their fresh weights were documented. Samples were digested with HClO_4_ (3 ml) and H_2_O_2_ (1 ml) at 25 °C for about 2 d with intermittent shaking till the solution turned colourless. Digestion mixture (300 µl) was added to the scintillation cocktail (3.5 ml) and incubated for 4 h with vigorous shaking at room temperature. ^32^Pi radioactivity was determined in both the root and shoot by using a liquid scintillation counter (Tri-Carb 2100 TR, Perkin Elmer). ^32^Pi counts in the root were used for determining the uptake rate, while that in the root and shoot were used for computing the distribution (shoot/root).

### Statistical analysis

Data were collected from 2–3 independent biological experiments and analyzed for significant differences using IBM SPSS Statistics 20 program.

## Electronic supplementary material

Supplementary Information
