# Supplementary material for: OsSIZ2 exerts regulatory influences on the developmental responses and phosphate homeostasis in rice
Source: Sci Rep. 2017 Sep 25;7:12280. doi: 10.1038/s41598-017-10274-5 (PMC5612973; doi:10.1038/s41598-017-10274-5)

# *OsSIZ2* exerts regulatory influences on the developmental responses and phosphate homeostasis in rice

Wenxia Pei^1^, Ajay Jain^2^, Yafei Sun^1^, Zhantian Zhang^1^, Hao Ai^1^, Xiuli Liu^1^, Huadun Wang^1,3^, Bing Feng^1^, Rui Sun^1^, Hongmin Zhou^1^, Guohua Xu^1^ & Shubin Sun^1^*

^1^State Key Laboratory of Crop Genetics and Germplasm Enhancement, Key Laboratory of Plant Nutrition and Fertilization in Low-Middle Reaches of the Yangtze River, Ministry of Agriculture, Nanjing Agricultural University, 210095, China

^2^Amity Centre of Nano Biotechnology and Plant Nutrition, Kant Kalwar, NH-11C, Jaipur -303002, India

^3^Present address: Provincial Key Laboratory of Agrobiology, Jiangsu Academy of Agricultural Sciences, Nanjing, China

Correspondence and requests for materials should be addressed to S.B.S (sunshubin@njau.edu.cn)


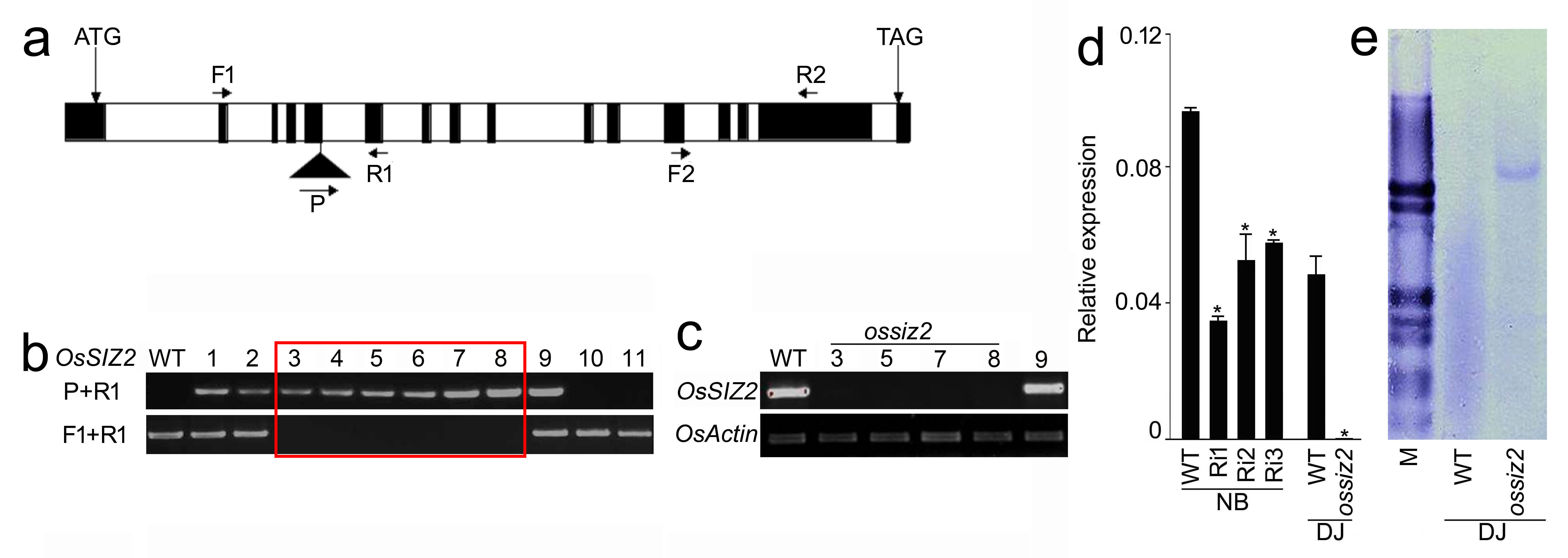


**Figure S1. Isolation and validation of *OsSIZ2* mutants.** (**a**) Schematic representation of *OsSIZ2* showing distribution of exons (black box) and introns (white box) and the location of T-DNA insert (black triangle). (**b**) The set of primers (P+R1 and F1+R1) was used for identifying homozygous *ossiz2* knockout mutants indicated by red box. (**c**) Semi-quantitative RT-PCR analysis was employed for determining the transcript abundance *OsSIZ2* in the WT (DJ), homozygous (lines 3,5,7 and 8) and heterozygous (line 9) mutants. (**d**) qRT-PCR analysis of the relative expression levels of *OsSIZ2* in the WTs (NB and DJ) and the mutants (Ri1-3 and *ossiz2*). *OsActin* was used as an internal control. Values are means ± SD (*n* = 3). Asterisks indicate that the values differ significantly (*P* < 0.05) in the mutants compared with their corresponding WT. (**e**) Southern blot analysis of the copy number in *ossiz2* in DJ background. M, Marker.

**Table S1** List of primers used for gDNA PCR, semi-quantitative RT-PCR and qRT-PCR

| **Primers used for the identification of homozygous *ossiz2* mutants** | | |
| --- | --- | --- |
| P | AACGCTGATCAATTCCACAG | |
| R1 | TCTCTCAATTTTGGCCAAGC | |
| F1 | TCACCAAAGGTTAGCAGCAAC | |
| **Primers used for semi-quantitative RT-PCR** | | |
| Gene | Locus ID | Primers |
| *OsSIZ2* | LOC_Os03g50980 | F2: TGGAACCGAGGCAGATAA |
|  |  | R2: TGTTGGTGCTGGAGGAAA |
| *OsActin* | LOC_Os03g50885 | F: GGAACTGGTATGGTCAAGG |
|  |  | R: AGTCTCATGGATACCCGCAG |
| **Primers used for qRT-PCR** | | |
| Gene | Locus ID | Primers |
| *OsSIZ1* | LOC_Os05g03430 | F: AAAGCTGCCATCCAGAAATCAT |
|  |  | R: AGCTTGTAAACCATTGAGGCAAC |
| *OsSIZ2* | LOC_Os03g50980 | F: ATGGCACGGCAGGTTTAGAC |
|  |  | R: TGGTGGCATCTCTCCTCTCAA |
| *OsPHR2* | LOC_Os07g25710 | F: GACCAGAATTGTCTGAAGGTTCTT |
|  |  | R: ACGCAATGCCTCAGTGAGAT |
| *OsIPS1* | LOC_Os03g05334 | F: TTGGCAATTATTCGGTGGAT |
|  |  | R: ACCATTTCACCATCCTCTTTATG |
| *OsPT1* | LOC_Os03g05620 | F: CGCTTCCGTACGAGTGGTAGT |
|  |  | R: GGTTCTTTCAAATCCAGGGAAA |
| *OsPT2* | LOC_Os03g05640 | F: GACGAGACCGCCCAAGAAG |
|  |  | R: TTTTCAGTCACTCACGTCGAGAC |
| *OsPT8* | LOC_Os10g30790 | F: AGAAGGCAAAAGAAATGTGTGTTAAAT |
|  |  | R: AAAATGTATTCGTGCCAAATTGCT |
| *OsPAP10a* | LOC_Os01g56880 | F: ATACTGGCAGCCGACGGATGA |
|  |  | R: GAGGGAGCTGGAGCGGAGAA |
| *OsSQD2* | LOC_Os01g04920 | F: CTGAAAACGGTAATGGATAGG |
|  |  | R: AACAACAACAGCACGAGC |
| *OsPHO1;2* | LOC_Os02g56510 | F: GTTCACGGAATGGTAATGGGACA |
|  |  | R: CGACTTCCATGGCGAGAT |
| *OsmiR399j* | MI0001062 | F: GGAGCATGTAAGTCTTTTGTAGC |
|  |  | R: GGCAACTCTCCTTTGGCAGA |
| *OsActin* | LOC_Os03g50885 | F: CAACACCCCTGCTATGTACG |
|  |  | R: CATCACCAGAGTCCAACACAA |

Original Image of Figure S1b.

1. Two-round PCR was employed for identifying homozygous *ossiz2* knockout mutants by using T-DNA right border-specific primer P and *OsSIZ2*-specific primer R1 (the red box was the cropped gels/blots “P+R1” in Figure S1b).
2. Two-round PCR was employed for identifying homozygous *ossiz2* knockout mutants by using *OsSIZ2*-specific primers F1 and R1 (the red box was the cropped gels/blots “F1+R1” in Figure S1b).


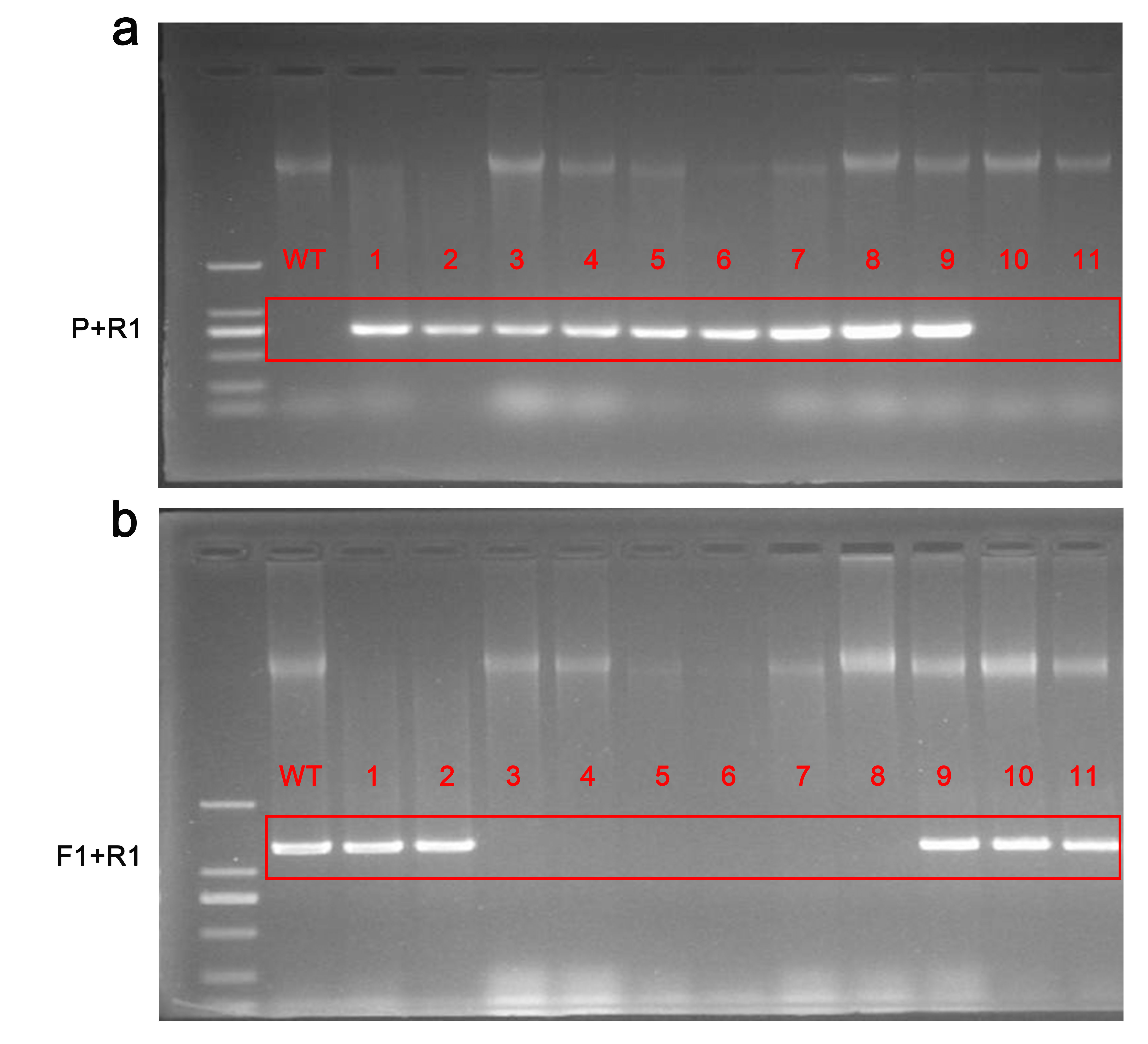


Original Image of Figure S1c

Semi-quantitative RT-PCR by using F2 and R2 primers for validating the lack of *OsSIZ2* transcripts in *ossiz2* mutants.

1. The expression of *OsActin* in WT and *ossiz2* mutants (the red box was the cropped gels/blots “*OsActin*” in Figure S1c).
2. The expression of *OsSIZ2* in WT and *ossiz2* mutants (the red box was the cropped gels/blots “*OsSIZ2*” in Figure S1c).


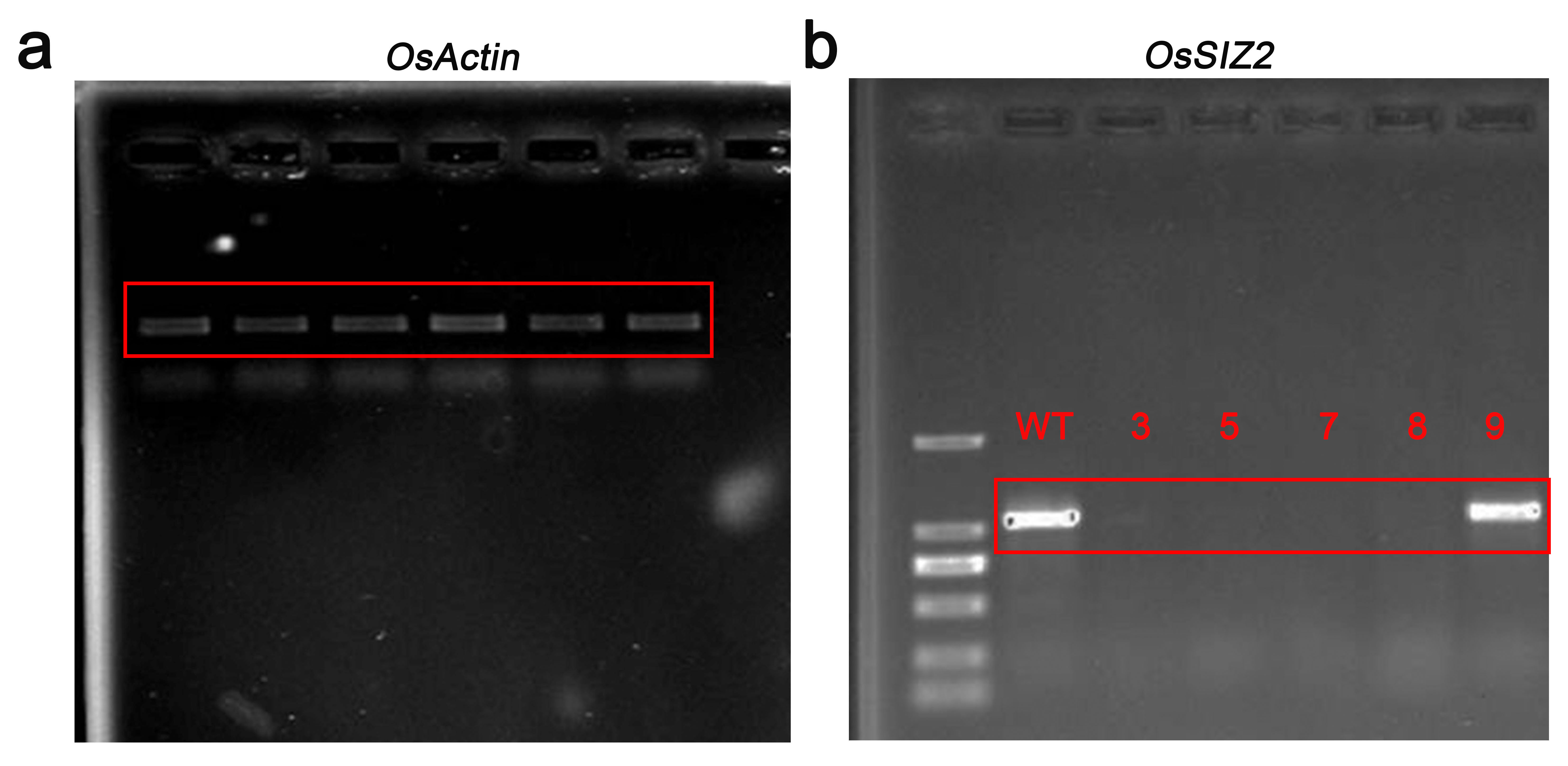

Supplement: Supplementary file 1 — Supplementary Information [file 41598_2017_10274_MOESM1_ESM.doc]
